# Near-Infrared Spectroscopy as a Diagnostic Tool for Distinguishing between Normal and Malignant Colorectal Tissues

**DOI:** 10.1155/2015/472197

**Published:** 2015-01-13

**Authors:** Hui Chen, Zan Lin, Lin Mo, Tong Wu, Chao Tan

**Affiliations:** ^1^Yibin University Hospital, Yibin, Sichuan 644000, China; ^2^Key Lab of Process Analysis and Control of Sichuan Universities, Yibin University, Yibin, Sichuan 644000, China; ^3^The First Affiliated Hospital, Chongqing Medical University, Chongqing 400016, China; ^4^The Affiliated Hospital, North Sichuan Medical College, Nanchong, Sichuan 637000, China

## Abstract

Cancer diagnosis is one of the most important tasks of biomedical research and has become the main objective of medical investigations. The present paper proposed an analytical strategy for distinguishing between normal and malignant colorectal tissues by combining the use of near-infrared (NIR) spectroscopy with chemometrics. The successive projection algorithm-linear discriminant analysis (SPA-LDA) was used to seek a reduced subset of variables/wavenumbers and build a diagnostic model of LDA. For comparison, the partial least squares-discriminant analysis (PLS-DA) based on full-spectrum classification was also used as the reference. Principal component analysis (PCA) was used for a preliminary analysis. A total of 186 spectra from 20 patients with partial colorectal resection were collected and divided into three subsets for training, optimizing, and testing the model. The results showed that, compared to PLS-DA, SPA-LDA provided more parsimonious model using only three wavenumbers/variables (4065, 4173, and 5758 cm^−1^) to achieve the sensitivity of 84.6%, 92.3%, and 92.3% for the training, validation, and test sets, respectively, and the specificity of 100% for each subset. It indicated that the combination of NIR spectroscopy and SPA-LDA algorithm can serve as a potential tool for distinguishing between normal and malignant colorectal tissues.

## 1. Introduction

Nowadays, cancer has become one of the principal causes to death of diseases [1, 2]. Great efforts have been paid for various cancer-related researches. Cancer diagnosis has become the central topic of research in cancer treatment. The conventional methods for cancer diagnosis are mainly based on the morphological appearance of the tumor tissue. The limitations for this method are the strong bias in discriminating the tumor by pathology expert and also the difficulties of differentiating between cancer subtypes [3].

Colorectal cancer is a disease of genes that control the proliferation, differentiation, and death of colon cells [4]. It has become the fourth most common cancer and continues to be the third leading cause of cancer-related deaths in both men and women, accounting for about 10% of all cancer deaths annually [5]. If colorectal cancer is found during its early stages, the 5-year relative survival rate is 90%. However, only about one-third of colorectal cancers are detected at early stages [6]. Although there are some available methods for diagnosing colorectal cancer [7], for example, serum markers, flexible sigmoidoscopy, and colonoscopy, the final result still relies on the gold standard of histopathologic diagnosis, which is time-consuming and strongly dependent on the pathologist's subjective judgment and experience. Hence, there is an urgent need to develop simple and fast diagnostic methods.

Recent researches have demonstrated the applicability of optical spectroscopic technique for fast, noninvasive, and in situ diagnosis of various diseases including cancer. Infrared (IR) and near-infrared (NIR) spectroscopy especially have been proved to be useful tools for disease diagnosis because of their potential to probe the changes of tissues and cells at the molecular level [8]. It is known that the generation and progression of any cancers manifest themselves at the molecular level before morphologic changes emerge, which cannot be detected by traditional methods or even pathologic examinations [9]. NIR spectroscopy, as a powerful tool with practical advantages, can rapidly capture the information of chemical bonds in function groups and is therefore sensitive to changes in molecular composition and structures [10–12]. Cancer tissues differ from the normal ones in the compositions and any alterations in the compositions of the tissues can be probed and used for diagnostic purposes. NIR technique has been used in several cancer researches such as lung [13], gastric [14], esophagus [15], endometrial [16], and pancreatic [17].

However, the NIR spectrum mainly corresponds to overtones and combinations of fundamental vibration transitions that occur in the IR region and is therefore overlapping, broad, and weak and without distinct signature of individual components [18]. A NIR-based diagnostic application requires a suitable diagnostic model that can best discriminate the measured spectra from an unknown tissue. Over the years, a variety of modeling algorithms have been developed or used for optical diagnosis of cancer. Both traditional algorithms such as soft independent modeling of class analogy (SIMCA) [19] and novel algorithms such as support vector machine (SVM) [20] have been used for this purpose. The NIR spectrum comprises measurements over a large number of channels for each sample. In many cases, the responses of different instrument channels exhibit strong correlation and there exist some channels without relevant information. Thus, it is beneficial to use only a subset of channels rather than the entire set of measurements [21]. Also, such a step facilitates the interpretation of the model and is useful to guide the design of less costly instruments. Recent efforts are directed towards using variable selection to identify the best diagnostic features for obtaining a simple and easily interpreted model. In this context, Araújo et al. [22] developed the successive projection algorithm (SPA) for selecting variables in multiple linear regressions (MLR). In a subsequent work, Pontes et al. [23] extended the basic SPA to handle classification problems by merging with linear discriminate analysis (LDA), which results in the so-called SPA-LDA method. SPA-LDA has been successfully applied in various classification tasks such as coffee and soil classification [24, 25].

The present paper proposed an analytical strategy for distinguishing between normal and malignant colorectal tissues by combining the use of NIR spectroscopy with variable selection. For this purpose, the SPA-LDA was used to seek a reduced subset of variables/wavenumbers and build a diagnostic model of LDA. For comparison, the partial least squares-discriminant analysis (PLS-DA) based on full-spectrum classification was also used as the reference. Principal component analysis (PCA) was used for a preliminary analysis. A total of 186 spectra from 20 patients with partial colorectal resection were collected and divided into three subsets for training, optimizing, and testing the model. The results showed that, compared to PLS-DA, SPA-LDA provided a simpler and better model, which used only three wavenumbers/variables (4065, 4173, and 5758 cm^−1^) to achieve the sensitivity of 84.6%, 92.3%, and 92.3% for the training, validation, and test set, respectively, and the specificity of 100% for each subset. It indicated that the combination of NIR spectroscopy and SPA-LDA algorithm can serve as a potential tool for distinguishing between normal and malignant colorectal tissues.

## 2. Theory and Methods

### 2.1. Partial Least Squares-Discriminant Analysis (PLS-DA)

Partial least squares (PLS) regression is a classic latent variable-based multivariate calibration method. Partial least squares-discriminant analysis (PLS-DA) is a classification algorithm that combines the properties of PLS regression with discriminant analysis [26]. The outstanding advantage of PLS-DA is that the main sources of variability in the dataset are modeled by the so-called latent variables (LVs), therefore, in the associated scores and loadings, making easy the visualization and understanding of data structure and relations in the dataset. Actually, PLS-DA is a special form of PLS modeling and focuses on finding the variables and directions in multivariate space, which discriminates the known classes in the training set. If there are only two classes to separate, the PLS model uses one dummy variable, which codes for class membership as follows: 1 for samples belonging to a given class of interest and 2 for samples belonging to a different class. A discriminant model is developed by regression of the independent matrix (spectral data) on the assigned dummy variable.

The model constructed on the experimental dataset can be used to assign unknown samples to a previously defined class based on its measured features such as spectrum. Classification of a new sample is derived from the output value of the PLS model. The output value is a real number, instead of an integer, which should ideally be close to the values used to codify the class (either 1 or 2). A threshold between 1 and 2 is set so that a sample is assigned to class 1 if the predicted value is smaller than the threshold or assigned to class 2 if the predicted value is above the threshold. PLS-DA uses the appropriate number of LVs, that is, linear combinations of the original variables, to maximize the discrimination among the classes. The number of LVs can be optimized by the criterion of lowest prediction error in cross validation.

### 2.2. Successive Projection Algorithms-Linear Discriminant Analysis (SPA-LDA)

The successive projections algorithm (SPA) is a forward variable selection method aimed at minimizing variable collinearity in modeling. It was originally developed by Araújo et al. in the context of multivariate calibration [22]. In SPA, the selection of variables is formulated as a combinatorial optimization problem with constraints. The optimization is restricted to certain subsets of variables, which are the results of a sequence of projection operations related to the matrix of instrumental responses. Therefore, the times of evaluating cost function are considerably reduced compared to an exhaustive search. In multivariate calibration, SPA is aimed at screening variables for building multiple linear regression (MLR) models. Subsequently, SPA has been extended to improve the performance of classification models of linear discriminant analysis (LDA), which easily suffered from multicollinearity among the input variables.

**Figure 1:**
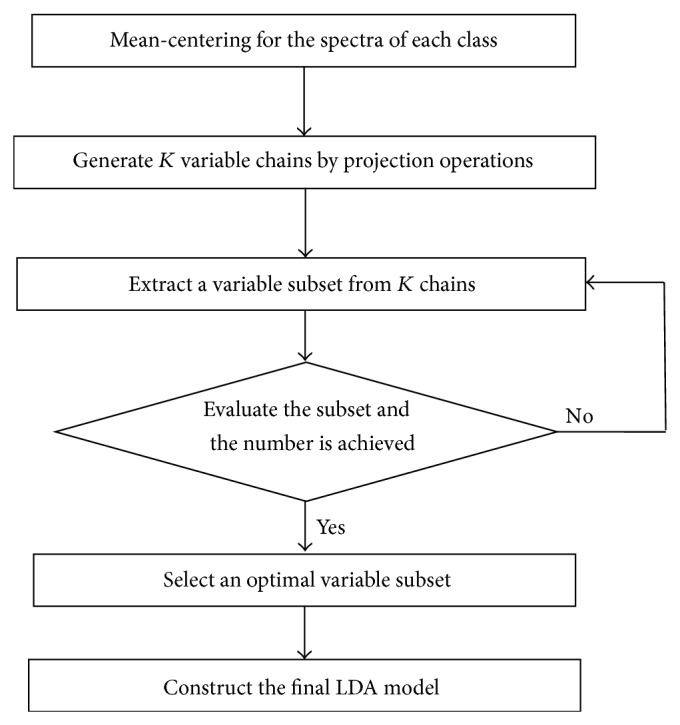
The flowchart of the SPA-LDA algorithm.

The combination of SPA and LDA is expressed as SPA-LDA.  Figure 1 gives the flowchart of the SPA-LDA algorithm. SPA-LDA focuses at selecting a subset of variables with minimal collinearity and appropriate discriminating ability for use in classification problems. For this purpose, it is assumed that a training set consisting of *N* samples with known class labels is available for guiding the process of variable selection. In the case of spectroscopic dataset, each sample consists of a spectrum with *K* points (wavenumbers/wavelengths). The SPA-LDA scheme comprises two main phases [27]. In Phase 1, the *N* training samples/spectra are first centered on the mean of each class and stacked in the form of a matrix *X*  (*N* × *K*). Each column of *X* corresponds to a variable. Projection operations related to the columns of *X* are then carried out to create *K* chains with *L* variables. Due to the loss of freedom degrees in the process of calculating class means, the chain length is limited by *N* − *C*, where *C* is the number of classes involved in the problem. Each time, the chain is initialized by one of the available *K* variables. Subsequent variables are selected to the chain in order to display the least collinearity with the previous ones. The collinearity is evaluated by the correlation between the respective column vectors of *X*. In Phase 2, different variable subsets are extracted and evaluated. For each of the *K* chains formed in Phase 1, a total of *L* subsets of variables can be extracted by using one up *L* variables in the order in which they are selected. Thus, a total of *K* × *L* subsets of variables can be generated. These candidate subsets are assessed in terms of a cost function involving the average risk of misclassification over the validation set. The cost function is defined as

(1) $$\begin{array}{c} G_{\text{cost}}=\frac{1}{N_{\text{val}}}\overset{N_{\text{val}}}{\underset{n=1}{\sum }}g_{n}, \end{array}$$

where

(2) $$\begin{array}{c} g_{n}=\frac{{\text{MD}}^{2}\left[x_{\text{val},n},\overset{-}{x}\left(I_{n}\right)\right]}{\min_{I_{j}\neq I_{n}}{\text{MD}}^{2}\left[x_{\text{val},n},\overset{-}{x}\left(I_{j}\right)\right]}. \end{array}$$

In (2), the numerator ${\text{MD}}^{2}\left[x_{\text{val},n},\overset{-}{x}\left(I_{n}\right)\right]$ is the squared Mahalanobis distance between the *n*th validation sample **x**_val,*n*_ and the center of its true class calculated over the training set by the formula:

(3) MD2xval,n,x−In=xval,n−x−InS−1xval,n−x−InT,

where **S** is a pooled covariance matrix calculated on the training set, instead of using a separate estimate for each class. To have a well-posed problem, the number of training samples should be larger than the number of variables included in a LDA model; otherwise, the estimated **S** will be singular, which makes it impossible to calculate the matrix inverse. The denominator in (2) corresponds to the squared Mahalanobis distance between **x**_val,*n*_ and the mean of the nearest wrong class. A small value of *g*_*n*_ indicates that **x**_val,*n*_ is close to the center of its true class and distant from the centers of all other classes. The cost function is defined as the average of *g*_*n*_ of all samples in the validation set. So, the minimization of the cost function can lead to better separation of samples of different classes.

Once the variables have been selected, a SPA-LDA model can be obtained. For a new sample, its Mahalanobis distance with respect to the mean vector of each class can be calculated and it can then be assigned to the class for which its Mahalanobis distance is the smallest.

## 3. Experimental

### 3.1. Preparation of Colorectal Tissue Samples

Colonic tissue samples were collected from 20 patients who underwent partial colorectal resection at the Affiliated Hospital of North Sichuan Medical College and the First People's Hospital of Yibin of China. All patients were histopathologically proven malignancies of the colon. After surgical resections, the tissue samples were immediately fixed in 10% formalin solution and then stored in the laboratory for spectral measurements. To ensure that the NIR spectra were representative of the pathology, the peer tissues were processed as paraffin embedded blocks for pathologic confirmation. The average age of the patients was 54 years with the youngest being 31 years and the oldest being 71 years. The study had been approved by the local ethics committee and the consent for using the tissue samples was obtained. It was believed that the positions with 5–10 cm distance from the tumor were healthy and each site was also confirmed by experienced pathologist. A total of 186 NIR spectra from different sites of colonic tissue specimens were acquired, in which 78 spectra were from cancerous positions and 108 spectra from normal positions. Different spectra correspond to different positions. All spectra were divided into three subsets: the training set, the validation set, and the test set. Each subset consisted of 26 cancerous and 36 normal spectra from different patients. For classification purposes, each spectrum was assigned a class label (1 for cancer and 2 for normal). The training and validation sets were used in the modeling procedures whereas the test set was only used in the evaluation and comparison of the final classification models.

### 3.2. Instrument and Spectrum Acquisition

The FT-NIR spectrometer of Antaris II (Thermo Fisher Scientific, USA) equipped with an InGaAs detector and a fiber-optic probe (SabIR) was used in this work for spectra collection. The SabIR is a high-performance optical probe able to perform remote nondestructive sampling. The measurement was done in diffuse reflectance mode. The outer and light spot diameters of the probe were about 20 mm and 3 mm, respectively. Thus, during each measurement, the measured area was appropriately 7.0 mm^2^. The spectrometer was controlled by the accompanied Result 3.0 software. Each spectrum was taken as an average of 32 successive scans from 4000 to 10000 cm^−1^ with spectral resolution of 4 cm^−1^. The record format of spectrum was Log⁡ (1/*R*), where *R* was the sample diffuse reflectance. To minimize the influence of tissue size and thickness, each individual spectrum was preprocessed by the standard normal variate (SNV) and first derivative. The SPA algorithm was implemented by the software of SPA_GUI provided by Araújo. All preprocessing and other calculations were performed by homemade codes in MATLAB 7.0 for Windows.

## 4. Results and Discussion

### 4.1. Spectral Band Assignment

**Figure 2:**
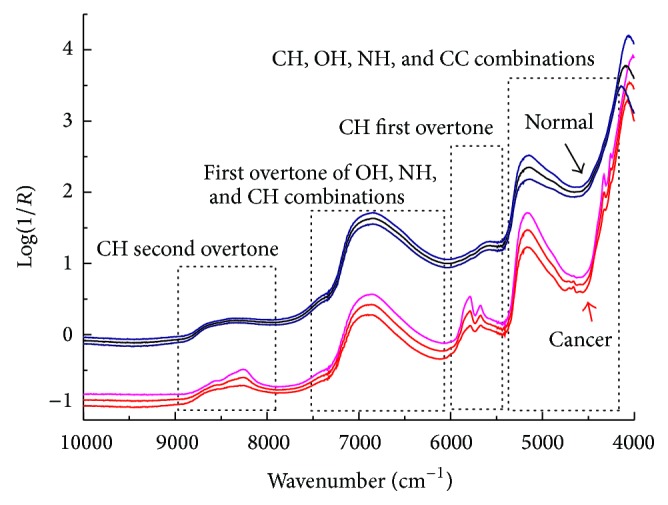
Populations mean spectra and the standard deviation of cancerous and normal tissue specimens.

Figure 2 shows the populations mean spectra and the standard deviation of cancerous and normal tissue specimens. Spectra from cancerous tissues were different in some regions, which reflected certain changes in the levels of various biochemical compositions due to canceration. It is also clear in Figure 2 that the spectral peaks of raw NIR signal are broad and overlapping and therefore make it impossible to carry out direct quantification analysis. Even if NIR spectrum cannot provide significantly different peaks like midinfrared (MIR) spectroscopy, it still includes much information on chemical composition of the tissue. Also provided in Figure 2 were the assignments of bands to different chemical substructures. From left to right in the region of 10000–4000 cm^−1^, four subregions correspond to the CH, NH, OH, and CC combinations, CH first overtones, first overtone of OH, NH, and CH combinations, and CH second overtone of fundamental vibration transitions, respectively. These NIR spectroscopic bands coupled with unsupervised pattern recognition have also been used for gastric cancer differentiation [14]. Each NIR spectrum is actually a mixture of the spectral signatures of various tissue components, especially proteins, lipids, and carbohydrates. The differences in composition between cancerous and normal tissues have been extensively investigated by chemical, histochemical, and biochemical means. For example, carbohydrate level was reduced in cancer tissues compared to normal tissues. The phosphate content of normal tissues was higher than cancerous ones. Overall, the NIR spectrum can provide information on tissue blood flow, oxygen saturation and consumption, and compositions. Thus, any alterations in the composition of the tissues can be captured in NIR spectrum and used for diagnostic purpose. It is also noteworthy that the spectral profile variation in some regions is higher for the cancerous tissues. It is maybe due to different stages of carcinogenesis of the tissues and differences in the thickness of the tissues, which influence spectral reflectance caused by photon penetrating depths.

### 4.2. Principal Component Analysis

**Figure 3:**
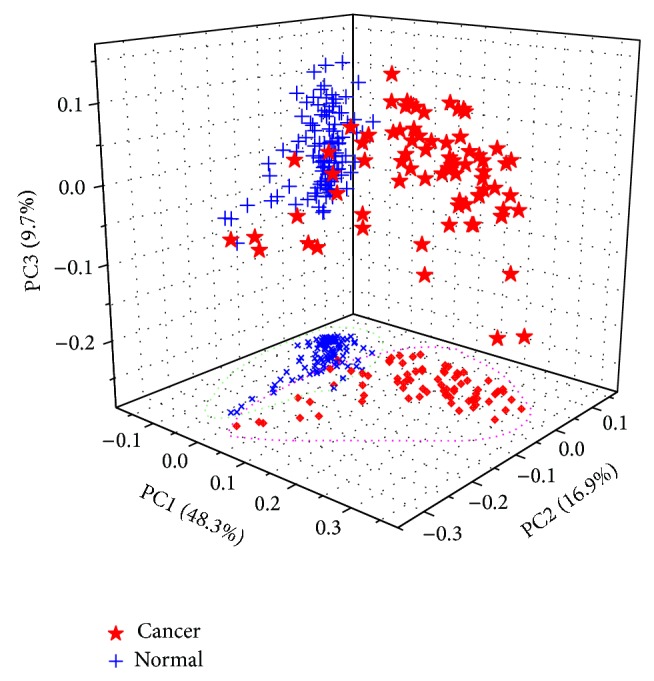
Three-dimensional scatter plot of the first three principal components (PCs) and its 2-dimensional projection. The variance explained by each PC is indicated in parenthesis.

Principal component analysis (PCA) was used to examine the possible clustering in samples and investigate the extent to which NIR features can differentiate cancerous and normal tissues.  Figure 3 provided the three-dimensional scatter plot of the first three principal components (PCs) and its 2-dimensional projection. The first three PCs accounted for about 80% of the total variation in the NIR spectra. As can be seen in Figure 3, the separation was not clear and there existed some overlaps between cancerous and normal samples, implying that the data structure or relationship was maybe complex and nonlinear. Therefore, to determine whether a tissue is cancerous or not from its NIR spectrum, a mathematical model needs to be trained by using some known samples.

### 4.3. Model Construction and Optimization

Both the PLS-DA and SPA-LDA algorithms were used for constructing the diagnostic models.

**Figure 4:**
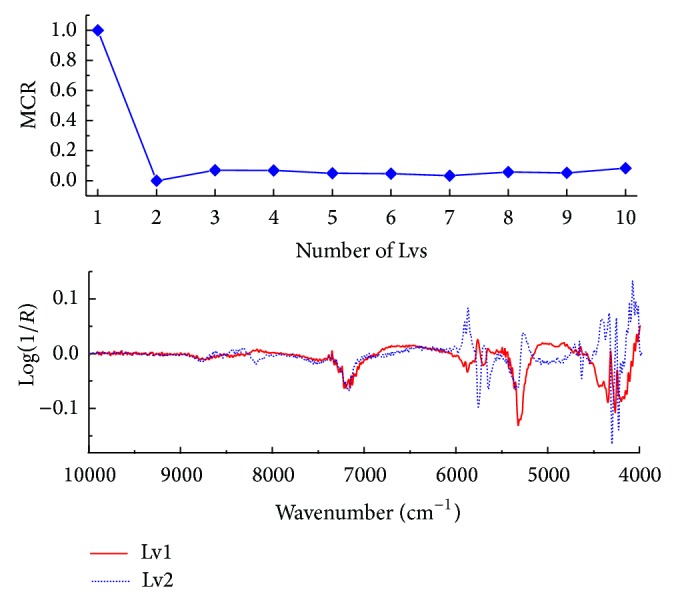
The influence of the number of latent variables (LVs) in the PLS-DA model on the misclassified ratio (MCR) and the first two loading vectors.

**Figure 5:**
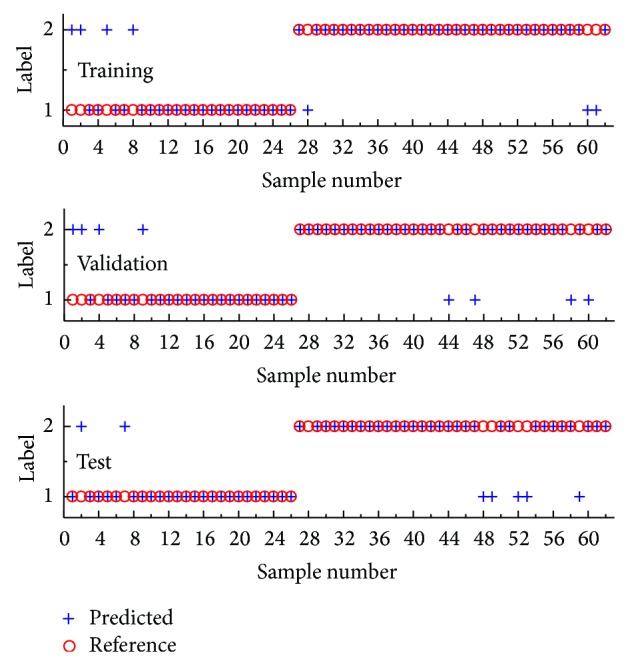
The prediction performance of the final PLS-DA model on different subsets.

When the PLS-DA model was constructed, one major issue was the choice of the optimal number of latent variables (LVs), which was carried out by a 5-fold cross validation procedure. When performing cross validation, the samples in the training set were first divided into five cross validation groups, that is, cancellation groups. Each cancellation group was first assigned 5 cancerous spectra and 7 normal spectra and the remaining spectra entered into the fifth group. Each cross validation group was removed from the training set, one at a time. Each time, the model was trained on the remaining samples and then used to predict the samples in the cross validation group.  Figure 4 illustrated the influence of the number of LVs in the PLS-DA model on the classification error (Err.). It seemed that the minimum misclassification ratio corresponded to 2 LVs, meaning that a relatively simple classification model was obtained, that is, a model based on few latent variables, which was preferable in terms of both model interpretation and stability. Considering that the loading can offer the possibility of observing the importance of features, the loading vectors of the selected LVs were also provided in Figure 4. Clearly, the LV1 and LV2 focused on the CH first overtones and CH, NH, CH, and CC combinations regions, respectively.  Figure 5 showed the prediction performance of the final PLS-DA model on the training, validation, and test sets. For either the training or test set, seven spectra were misclassified. The sensitivity and specificity were 84.6%, 84.6%, and 91.7% and 92.3%, 88.9%, and 86.1% for the training, validation, and test sets, respectively.

**Figure 6:**
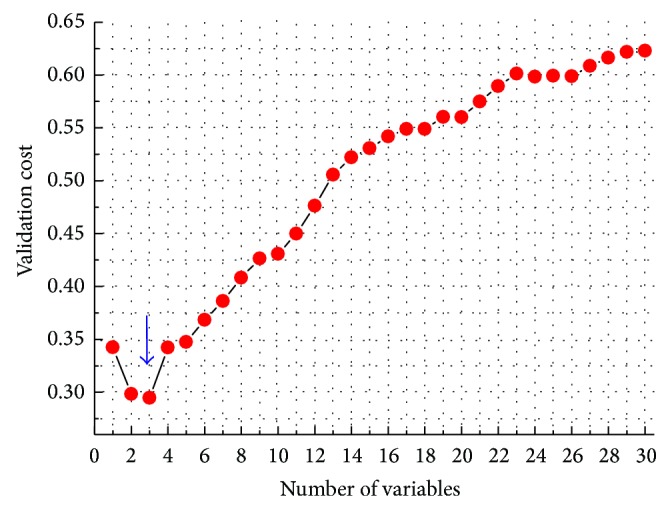
Validation cost (*G*_cost_) as a function of the number of variables selected by SPA-LDA algorithm. The arrow indicates the minimum point of the cost curve, which occurs at three wavenumbers.

**Figure 7:**
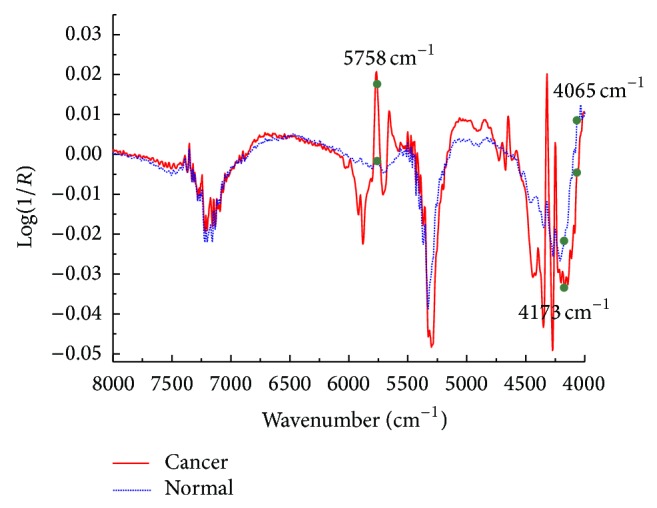
Preprocessed mean spectra of cancerous and normal tissues by 1st derivative in the range of 8000–4000 cm^−1^. The solid circle markers indicate the positions in the spectra of the wavenumbers selected by SPA-LDA algorithm.

**Figure 8:**
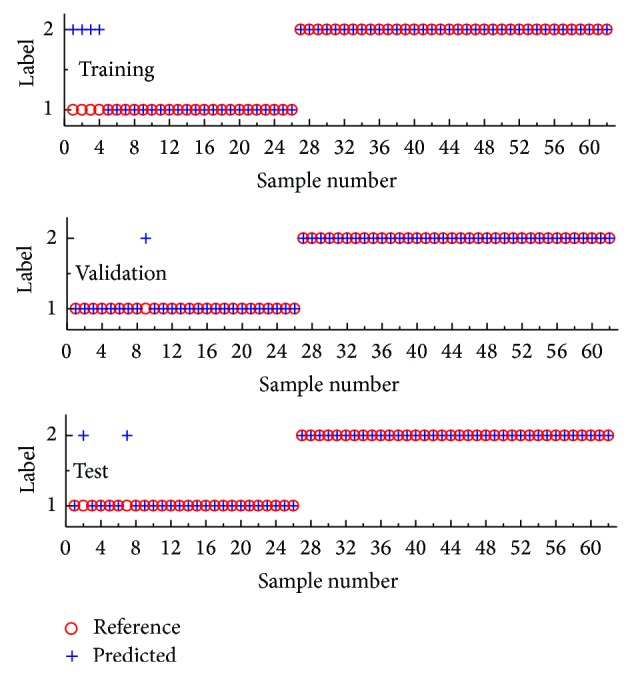
The prediction performance of the final SPA-LDA model on different subsets.

The SPA-LDA modeling resulted in only three variables/wavenumbers, which correspond to the minimum point of the validation cost curve, as the arrow indicated in Figure 6.  Figure 7 gave the preprocessed mean spectra of cancerous and normal tissues by 1st derivative in the range of 8000–4000 cm^−1^, where the solid circle markers indicated the wavenumber positions selected by SPA-LDA. As can be seen in Figure 7, the selected three wavenumbers (4065, 4173, and 5758 cm^−1^) are indeed related to these characteristic points such as spectral peaks and shoulders. Similarly, Figure 8 showed the prediction performance of the final SPA-LDA model on the training, validation, and test sets. There existed 4, 1, and 2 misclassified spectra for the three sets, respectively. The sensitivity was 84.6%, 92.3%, and 92.3% for the training, validation, and test set, respectively. The specificity for each subset was the same, that is, 100%. It was clear that SPA-LDA used only three variables to achieve superior performance to PLS-DA. Why only three variables lead to better model is maybe ascribed to the fact that the NIR signal strength between different channels is considerably correlated. Often, only a variable can represent the information distributed in its adjacent variables. Such a phenomenon is also in accordance with the purpose of SPA, which is to minimize collinearity among the selected variables. Moreover, SPA-LDA proves to be less sensitive to instrumental noise and more parsimonious than the other strategies.

## 5. Conclusions

The combination of NIR spectroscopy and two classification algorithms was evaluated in a study for distinguishing cancerous colon tissue from normal ones. The results showed that the SPA-LDA was preferable since it used only three single wavenumbers to achieve better performance than PLS-DA. The NIR technique has several advantages: it is inexpensive and less time-consuming and does not require special sample preparation. It can be applied in oncology, not only to diagnose cancerous tissue from normal tissue but also to understand basic process such as changes in metabolite concentration at the molecular level before histological manifestation. Based on more representative sample set, NIR is also expected to be used in grading of malignancies, which maybe remains our future work.
